# Clinical Characteristics of Preschool Children with Oppositional Defiant Disorder and Callous-Unemotional Traits

**DOI:** 10.1371/journal.pone.0139346

**Published:** 2015-09-29

**Authors:** Lourdes Ezpeleta, Roser Granero, Núria de la Osa, Josep M. Domènech

**Affiliations:** 1 Unitat d’Epidemiologia i de Diagnòstic en Psicopatologia del Desenvolupament, Grup de Recerca 2014 SGR 312 Generalitat de Catalunya, Barcelona, Spain; 2 Departament de Psicologia Clínica i de la Salut, Universitat Autònoma de Barcelona, Barcelona, Spain; 3 Departament de Psicobiologia i Metodologia de les Ciències de la Salut, Universitat Autònoma de Barcelona, Barcelona, Spain; University of Vienna, School of Psychology, AUSTRIA

## Abstract

There is a need to know whether callous-unemotional (CU) traits identify a more severe group of oppositional defiant children (ODD). The aim of this study is to ascertain cross-sectionally and longitudinally the specific contribution of CU levels and the presence of ODD in the psychological state of preschool children from the general population. A total of 622 children were assessed longitudinally at ages 3 and 5 with a semi-structured diagnostic interview and questionnaires filled out by parents and teachers. In multivariate models simultaneously including ODD diagnosis and CU levels, controlling by socioeconomic status, ethnicity, sex, severity of conduct disorder symptoms and other comorbidity, high CU scores were related to higher levels of aggression, withdrawn, externalizing and global symptomatology, functional impairment and higher probability of comorbid disorders and use of services. The contribution of CU traits on children’s psychological state was not moderated by the presence/absence of ODD. Stability for CU traits and number of ODD-symptoms between ages 3 and 5 was statistically significant but moderate-low (intra-class correlation under .40). Assessment and identification of CU traits from preschool might help to identify a subset of children who could have socialization problems, not only among those with ODD but also among those without a diagnosis of conduct problems.

## Introduction

Oppositional defiant disorder (ODD) is a highly prevalent condition [1] strongly comorbid with conduct disorder (CD) [2]. Research has frequently combined the two disorders in a single category (disruptive behavior disorders; DBD). Currently, it is proposed that ODD must be studied separately from CD, as the two disorders have different developmental trajectories and are associated with different risk processes [3].

Callous-Unemotional traits characterize a group of children with serious conduct problems, displaying a particular interpersonal and affective style distinguished by lack of empathy, lack of guilt and constricted emotional expression [4]. This concept has been associated mainly with CD. The DSM-5 [5] includes subtyping of conduct disorder considering the presence of CU traits on the basis that these traits identify a group of children with severely disordered conduct [6]. No subtypes based on CU traits have been identified for ODD.

On investigating CU traits, many studies have combined CD and ODD, blurring the specific associations each one may present. The combination of DBD and CU traits represents a quite severe scenario, involving higher levels of behavioral disinhibition (greater impulsivity-hyperactivity, reward-dominant response style, sensation seeking), more severe conduct problems (greater instrumental aggressive behavior, earlier-onset and more stable conduct problems, worse outcomes, poorer response to treatment), difficulties for processing cognitive stimuli (low sensitivity to punishment), reduced emotional responsiveness to fear and distress in others, specific temperamental characteristics (low levels of fear and of anxiety), compared to the case of DBD without CU [7]. Amygdala and orbitofrontal cortex anomalies, as described in the literature, could be underlying such difficulties [8]. The heritability of CU traits is estimated at between 40% and 78% [9]. The DBD-only group manifests higher reactivity to emotional and threatening stimuli, more intense reactivity to provocation, a hostile cognitive bias and low verbal intelligence, and has been more exposed to dysfunctional parenting practices [10]. Different deficits affect these two groups, so that conduct problems without CU may be associated with difficulties in emotional self-regulation, whereas conduct problems with CU is more likely to be related to difficulties in the development of conscience [11].

CU traits can appear very early in childhood [12]. These traits are not always associated with antisocial behavior, and it cannot be assumed that they always reflect “psychopathic” behavior [13]. Recent reviews have highlighted the need to clarify the relevance of CU traits over and above DBD [14]. Furthermore, little is known about CU as a classifier in ODD. Therefore, and in light of the next revision of classification systems such as the ICD, it is pertinent to ask whether CU traits also identify a subset of children with more severe behavioral problems when associated with ODD.

Relatively few studies have tackled the issue of CU traits in preschool children. It is in this period when abilities related to CU, such as empathy or guilt, which promote positive behavior and prosociality, begin to emerge [15], and when they could be more easily rectified in case of deviation. Several reports indicate the possibility of reliably assessing CU traits in preschoolers [16–18]. In very young children, high CU traits are associated with disorganized attachment [19], impaired eye contact [20], high aggression and problem behavior [12, 21], and inconsistent and harsh discipline [22–24]. Stability of high CU traits is associated with the poorest outcomes at follow-up [25]. Though children with high CU traits are less responsive to parenting training intervention [26], a range of studies have reported positive effects of interventions at these ages [27, 28]. For instance, Kochanska et al. [29] showed that CU traits moderate the effect between early parenting and externalizing behavior problems: in children high in CU traits, parents’ positive affect reduced the probability of future behavior problems. These associations highlight the importance of identifying CU traits early in life so as to promote more adaptive development.

Few studies have focused specifically on ODD to study its association with CU and to test for a relationship that may be clinically informative. In the research that has addressed this issue, in both clinical and community samples from middle childhood to adolescence, CU traits have shown significant correlation with a number of ODD symptoms [30, 31]. Regarding ODD and CU in preschoolers, Willoughby et al. [32] reported that CU traits were stable from ages 3 to 5, and distinguished a group of children with ODD+CU that were less fearful, recovered more easily after an upset, and showed less negative reactivity, lower heart period reactivity and higher levels of general arousal than those with ODD only. In a later study, Hawes et al. [33] found that children high in CU presented more severe ODD symptoms in comparison to those showing low CU.

Since CU traits have shown a certain stability from early childhood to adolescence [32, 34], and given the evidence of severe outcome associations, more research is needed in large community samples from different cultures to identify the clinical benefits of identifying the ODD plus CU traits subgroup, especially early in life, given the preventive potential of early identification. The goal of this study is to ascertain cross-sectionally and longitudinally: a) the specific contribution of the CU level and the presence of ODD diagnosis on psychological and functional measures as early as preschool age in the general population; b) the existence of a potential interaction CU×ODD, to determine if the contribution of CU levels on the children’s clinical state varied for children with ODD and those without the diagnosis; and c) the stability of CU traits from ages 3 to 5. In line with the previous literature on CD+CU in older children, and given that ODD is a disorder which also involves conduct problems related to social learning, we hypothesized that the presence of high CU trait scores early in life will contribute to an increase in psychological symptomatology and conditions associated with ODD, as well as with poorer prognosis. We also expect significant stability of the CU traits. There is a lack of research about the characteristics of children high in CU only [14]. However, it is expected that the presence of high scores in this trait early in life will alter the development of appropriate social cues and increase the likelihood of disruptive behavior.

## Method

### Participants

The data are from a large-scale longitudinal study of behavioral problems in preschool children starting at age 3 who were screened for behavior problems and followed-up annually until age 5 (the design procedure is detailed in [35]). The cross-sectional two-phase design involved first of all the selection of a random sample of 2,283 children from the census of preschoolers in grade P3 (3-year-olds) from Barcelona (Catalonia, Spain). A total of 1,341 families (58.7%) agreed to participate in the first phase of the study, of which 451 (33.6%) were of high socioeconomic status (SES) [36], 581 (43.1%) middle and 309 (23.3%) low. Children’s mean age was 3.0 years (*SD* = 0.18), 683 were boys (50.9%) and 1,198 (89.3%) were white. There were no sex differences (*p* = .95) between those who agreed to participate and those who declined, but semi-private schools were significantly more likely to refuse to participate than public schools (*p* < .001), and high SES families participated more than low-status families (*p* < .001).

In the second phase, all children with a positive screening score for behavioral problems and a random sample of 30% of children with a negative screening score were invited to continue. Cut-off for screen positive was a Strengths and Difficulties Questionnaire (SDQ^3-4^ [37]) score ≥ 4 on the conduct problems scale (cut-off corresponding to percentile 90 in community samples, considered the “abnormal band” scores) (see S1 Table) or a response option of 2 (“certainly true”) in any of the eight DSM-IV parent-reported oppositional defiant symptoms (four included in the SDQ^3-4^ conduct problem scale plus four items added from the DSM-IV definition of ODD). There were no differences in refusal to participate between the cohorts of positive screen (105 families declined, 20.1%) and negative screen (30 families, 12.8%) (*p* = .54). The final second-phase sample included 622 families (10.6% of those invited declined to participate in the second phase). No differences were found on comparing participants and refusals by sex (*p* = .82) or by type of school (*p* = .85). Children’s mean age was 3.0 (*SD* = 0.16), 311 were boys (50.0%) and 557 (89.5%) were white, while 210 (33.8%) were of high SES, 279 (44.9%) middle, and 133 (21.3%) low. Weighted DSM-IV prevalence in the final sample (*N* = 622) at age 3 was as follows: 3.7% attention deficit/hyperactivity disorder (ADHD), 6.9% ODD, 1.4% CD, 0.4% major depression, 3.0% minor depression, 2.2% separation anxiety, 3.7% specific phobia and 1.9% social phobia.

At age 5, 574 (92.3%) children continued in the study (ICU was available for *N* = 565 children, 90.8%). Participants and drop-outs at age 5 were statistically equal in sex (*p* = .238), SES (*p* = .127), baseline CU-trait mean (*p* = .311) and ODD diagnosis (*p* = .882).

Participating teachers from 54 schools had known the 3-year-olds for a mean of 7.6 months (*SD* = 2.2), and the five-year-olds for a mean of 11.6 months (*SD* = 6.2).

### Instruments

The *Diagnostic Interview for Children and Adolescents for Parents of Preschool and Young Children* (DICA-PPYC [38]) is a computerized semi-structured diagnostic interview for assessing the most common psychological disorders at ages 3–7 through algorithms, following the DSM-IV-TR criteria [39]. The assessment of the symptoms of each disorder is followed by questions about consultation and treatment received (use of services). The ODD diagnosis was used, together with ODD dimensions of irritability and headstrong nature derived from factor analysis with the symptoms of ODD [40]. Comorbidity was defined as the concurrence of other diagnoses (ADHD, CD, depressive disorders—major and minor depression–, and anxiety disorders—separation anxiety, generalized anxiety disorder, specific phobia and social phobia).

The interviews were answered mostly by the mothers (at age 3: 68.3% by mothers, 7.6% by fathers and 24.1% by both parents; at age 5: 74.3% by mothers, 8.6% by fathers and 17.1% by both).

The *Inventory of Callous-Unemotional Traits* (ICU [41]) includes 24 items coded on a 4-point Likert-type scale (0: *not at all true* to 3: *definitely true*) and covering three dimensions: Callousness, Uncaring and Unemotional. This instrument was responded to by the teachers on two occasions, when the children were 3 and 5 years old. Cronbach’s α for each scale of the age-3 and age-5 ratings were, respectively, .79 and.75 for Callousness, .88 and .89 for Uncaring, and .83 and .87 for Unemotional.

The *Children’s Aggression Scale* (CAS [42]) assesses aggressive behavior with 22 items on a 5-point Likert-type scale (0: *never* to 4: *many days*). Total score was used as a global index of aggressive behavior. This instrument was responded to by the teachers at ages 3 and 5. Cronbach’s alpha at ages 3 and 5, respectively, were .82 and .85 for the total score.

The *Relational aggression* measure was created for this research project. It contains 13 items using 5-point Likert-type scales (0: *never* to 4: *many days*) related to aggressive behavior in relationships with others (behaving unsociably, crying to get sympathy, being malicious, criticizing others behind their backs, being manipulative, being hurtful, ganging up with other children to isolate a child, etc.). Teachers answered the questionnaire at ages 3 and 5. Cronbach’s alphas were .90 at age 3 and .94 at age 5.

The *Social Communication Disorders Checklist* (SCDC [43]) is a one-dimensional 12-item scale for measuring social cognition as manifested in social and communication deficits in social reciprocity, non-verbal skills and pragmatic language usage. It was answered by the teacher when children were 5 years old (Cronbach’s alpha: .88).

The *Child Behavior Checklist* (CBCL/1½-5 [44]) measures behavioral and emotional problems through 100 items with 3 response options (0: *not true*, 1: *somewhat/sometimes true*, 2: *very true/often true*), and is answered by parents at ages 3 and 5. Cronbach’s alpha ranged from .41 (somatic complaints) to .92 (total score) at age 3, and from .46 (somatic complaints) to .92 (total score) at age 5.

The *Children’s Global Assessment Scale* (CGAS [45]) is a global measure of functional impairment filled out by the interviewer after the information has been obtained by parents in the diagnostic interview at ages 3 and 5. Scale scores range from one (maximum impairment) to 100 (excellent functioning). Scores over 70 indicate normal adjustment.

Teachers are familiar with children’s normative development and can observe the child in social situations. Moreover, their scores have shown higher internal consistency than those of parents, and have proved useful for discriminating CU traits in children [46]. Considering this, teachers were asked to report on CU traits, aggressive behavior, and social cognition.

### Procedure

The longitudinal project was approved by the ethics review committee of the Universitat Autònoma de Barcelona (Comissió d’Etica en l’Experimentació Animal I Humana, approval number CEEAH 1385). Informed written consent was obtained from parents of the children participating in the study, as approved by the ethics committee. Heads of the participating schools and parents were provided with a full description of the study. Families were recruited at the schools, and gave written consent. All parents of children from grade P3 at the participating schools were invited to answer the SDQ3-4, which was completed by families at home and returned to the schools. Families who agreed to participate and met the screening criteria were contacted by telephone and interviewed at the school. Interviewers (psychologists with master's degrees and psychology students supervised by two PhD-level clinical child psychologists) were previously trained and were blind to the children’s screening group. All interviews were audio-recorded and supervised. Weekly meetings were scheduled to monitor the cases, and the team members discussed the cases and difficulties with interview coding. After the interview with parents, interviewers rated the CGAS measure. Then parents answered the questionnaires and the teachers were asked to answer the questionnaires for completion before the end of the academic year.

### Statistical Analysis

Statistical analysis was carried out with SPSS20 for Windows. General Linear Models (GLM, for psychological quantitative measures) and logistic regressions (for binary measures) assessed the specific contribution of CU levels and the presence of ODD diagnosis on the psychological measures. These models were implemented in Complex Samples (CS) due to the multi-sampling design, defining a planning project with weights equal to the inverse probability of selection in the second phase of the design. For this modeling, the measures of CU (ICU-total raw score) and ODD (binary diagnosis present/absent) were considered as the independent variables and the analyses were adjusted by the covariates family SES, children’s sex and ethnicity, presence of comorbidities other than ODD and the number of DSM-IV CD symptoms. An initial model including the independent variables CU and ODD, their interaction CU×ODD and the covariates was tested. In the case of non-significant interaction (*p*>.05), this parameter was excluded and the main effects were estimated and interpreted for the CU and the ODD factors. For significant interaction, this parameter was retained in the models and single effects were estimated and interpreted. The analyses were carried out for the psychological measures at age 3 and for the outcomes at age 5.

The same statistical procedure (GLM and logistic regression into the CS system) also assessed the specific contribution of CU levels for the subsample of children diagnosed with ODD at baseline (*n* = 61). In these analyses, the CU raw score was considered as the independent variable and covariates were also SES, children’s sex and ethnicity, comorbidities other than ODD and the number of DSM-IV CD symptoms.

The increase in Type-I error due to multiple statistical comparisons was controlled through the Simes correction procedure [47], a corrective method which offers a more powerful test than the classic Bonferroni-correction. In addition, since from a practical-clinical perspective effect sizes are the most relevant objective of the analyses, and due to the fact that *p*-values are strongly dependent on sample size, all effect sizes for the relationships analyzed have been estimated by the confidence interval for the parameters, with the R^2^ measuring the global predictive capacity of the models (adjusted to the covariates).

Stability of the ODD-CU measures for ages 3 to 5 was estimated via the intra-class correlation coefficient (absolute agreement) for quantitative scores and kappa for categorical scores.

## Results

### Descriptive statistics for the psychological measures at ages 3 and 5

Table 1 contains the descriptives for the psychological measures analyzed in this study for children at ages 3 and 5 (mean and standard deviations for the quantitative variables and prevalences for binary outcomes).

**Table 1:** Descriptives for the variables of the study at ages 3 and 5.

	Age 3						Age 5					
	ODD = absent		ODD = present		Total		ODD = absent		ODD = present		Total	
	(*n = 561)*		(*n = 61)*		(*n = 622)*		(*n = 518)*		(*n = 56)*		(*n = 574)*	
	Mean	SD	Mean	SD	Mean	SD	Mean	SD	Mean	SD	Mean	SD
*ICU Callous-unemotional*												
Callousness	4.88	4.27	5.45	3.71	4.92	4.23	3.88	3.46	4.51	3.60	3.92	3.47
Uncaring	11.41	5.24	11.88	4.98	11.44	5.22	10.47	5.17	12.14	5.13	10.59	5.18
Unemotional	4.78	3.32	4.52	2.98	4.76	3.30	5.21	3.41	5.33	3.04	5.22	3.38
Total	21.13	10.08	21.93	9.27	21.19	10.02	19.58	9.59	22.00	9.60	19.75	9.60
*DSM-IV quantitative*												
Number ODD symptoms	0.84	1.16	4.61	0.86	1.10	1.49	0.85	1.31	2.36	2.11	0.95	1.43
ODD-irritability dimension	0.36	0.60	1.79	0.74	0.46	0.71	0.41	0.72	1.08	1.02	0.46	0.76
ODD-headstrong dimens.	0.48	0.76	2.82	0.77	0.64	0.96	0.44	0.77	1.18	1.26	0.49	0.83
Number CD symptoms	0.16	0.51	0.90	1.10	0.22	0.60	0.11	0.38	0.19	0.62	0.12	0.40
*Aggression*												
CAS Aggression: total	55.36	6.66	56.85	7.19	55.47	6.70	42.21	15.55	47.55	17.76	42.59	15.75
Relational aggression	46.40	13.29	49.67	13.82	46.63	13.34	42.51	17.57	46.04	18.95	42.76	17.68
* *Social cognition*												
SCDC: total (teacher)		---	---	---	---	---	2.68	3.83	3.37	3.88	2.72	3.83
*CBCL/1½-5 scales*												
Emotionally reactive	1.85	2.10	3.52	2.76	1.97	2.19	1.46	1.96	2.54	2.81	1.53	2.05
Anxious/depressed	2.27	2.02	3.95	2.67	2.39	2.11	1.89	1.94	2.70	2.20	1.95	1.97
Somatic complaints	1.60	1.67	2.11	1.95	1.63	1.70	0.98	1.37	1.60	1.62	1.02	1.40
Withdrawn	1.39	1.51	1.85	1.64	1.43	1.52	1.07	1.40	1.29	1.65	1.09	1.42
Sleep problems	2.82	2.73	4.38	3.06	2.93	2.78	1.60	2.03	2.20	2.39	1.65	2.06
Attention problems	1.96	1.72	2.92	2.25	2.03	1.77	1.50	1.72	2.18	2.09	1.54	1.76
Aggressive behaviour	8.01	4.81	15.59	5.06	8.54	5.19	5.49	4.83	9.70	7.01	5.78	5.11
Internalizing	7.12	5.62	11.38	7.18	7.41	5.84	5.41	5.29	8.13	6.97	5.59	5.46
Externalizing	9.98	5.79	18.51	6.18	10.57	6.21	6.99	5.87	11.88	8.30	7.32	6.18
Total	26.4	15.1	44.3	17.5	27.6	15.9	18.6	14.5	28.9	20.6	19.3	15.2
*Functional impairment*												
CGAS: total score	80.2	8.49	65.9	7.15	79.2	9.15	78.6	7.89	70.1	9.63	78.0	8.29
*Categorical measures*												
Comorbidity; *%*	1.8		9.9		2.3		2.5		6.3		2.7	
Use of services; *%*	16.0		45.1		18.0		14.4		31.3		15.5	

SD: standard deviation.

*Registered at age 4 (teachers’ report).

### Association between CU and ODD with psychological measures at age 3

Table 2 contains the GLM and logistic regressions assessing the contribution of the independent variables, CU levels, and the presence/absence of ODD on the children’s psychological measures for the total sample (*n* = 622), adjusted by the covariates family SES, children’s ethnicity and sex, other comorbid disorder different from ODD and the number of DSM-IV CD symptoms. All the variables entered in these models were registered at age 3. Firstly, the moderation effect CU×ODD was tested. All the interaction parameters obtained non-significant results (*p*>.05), indicating that the contribution of CU severity on the psychological measures considered in this study is statistically equal for children who presented ODD and those without the diagnosis (similarly, the contribution of the presence/absence of ODD on the clinical indicators is not moderated by CU levels). Due to the lack of significant interaction CU×ODD, these parameters were excluded from the modeling, and main effects were estimated and interpreted for CU and ODD factors. Main effects in these analyses provide the specific contribution of each of the factors (CU adjusted to ODD and ODD adjusted to CU) on the children’s clinical state. Results show that the higher the CU raw scores, the higher the levels of CAS-total aggression and relational aggression, the higher the levels of CBCL withdrawn, attention, aggressive, externalizing and total scores, the higher the probability of comorbid disorders and use of services, and the lower the level of CGAS functional impairment. The presence of ODD was related to high scores in the CBCL emotionally reactive, anxious/depressed, sleep problems, aggressive, internalizing, externalizing and total scales, and to low scores on CGAS and high probability of the presence of comorbid disorders and use of services.

**Table 2:** Association of the CU levels and the presence of ODD on the psychological measures at age 3 (total sample, n = 622).

Dependent variables	^1^CUx	Independent variables (entered simultaneously)												*R* ^2^
	ODD	CU raw score						Oppositional defiant disorder (ODD)						
	*p*	B	SE		95%CI (B)		* *p*	B	SE		95%CI (B)		* *p*	
*Aggression*														
CAS Aggression: total	.118	0.232	0.027		0.18;	0.28	**< .001**	-0.216	1.051		-2.28;	1.85	.837	0.110
Relational aggression	.671	0.586	0.054		0.48;	0.69	**< .001**	2.453	1.722		-0.93;	5.84	.286	0.177
*CBCL/1½-5 scales*														
Emotionally reactive	.239	0.009	0.010		-0.01;	0.03	.533	0.997	0.409		0.19;	1.80	**.026**	0.013
Anxious/depressed	.568	0.004	0.009		-0.01;	0.02	.680	1.077	0.381		0.33;	1.83	**.011**	0.015
Somatic complaints	.742	0.005	0.008		-0.01;	0.02	.665	0.311	0.288		-0.25;	0.88	.327	0.003
Withdrawn	.075	0.024	0.006		0.01	0.04	**.001**	0.124	0.233		-0.33	0.58	.595	0.023
Sleep problems	.298	-0.006	0.012		-0.03;	0.02	.680	1.386	0.431		0.54;	2.23	**.005**	0.015
Attention problems	.662	0.027	0.008		0.01;	0.04	**.004**	0.481	0.323		-0.15;	1.11	.192	0.025
Aggressive behaviour	.743	0.048	0.021		0.01;	0.09	**.049**	5.585	0.742		4.13;	7.04	**< .001**	0.071
Internalizing	.277	0.041	0.026		-0.01;	0.09	.109	2.447	1.022		0.44;	4.45	**.017**	0.014
Externalizing	.929	0.075	0.026		0.02;	0.13	**.003**	6.066	0.866		4.36;	7.77	**< .001**	0.065
Total	.407	0.141	0.068		0.01;	0.27	**.039**	11.938	2.408		7.21;	16.7	**< .001**	0.037
*Functional impairment*														
CGAS: total score	.063	-0.111	0.037		-0.18;	-0.04	**.003**	-10.03	1.028		-12.0;	-8.01	**< .001**	0.078
*Categorical measures*	*p*	Callousness: raw score (CU)						Oppositional defiant disorder (ODD)						*R* ^2^
		B	SE	OR	95%CI (OR)		* *p*	B	SE	OR	95%CI (OR)		* *p*	
Comorbidity	.689	0.065	0.016	1.07	1.03;	1.10	**< .001**	2.435	0.405	11.4	5.16;	25.3	**< .001**	.178
Use of services	.551	0.035	0.012	1.04	1.01;	1.06	**.003**	1.254	0.321	3.50	1.87;	6.58	**< .001**	.084

^1^p-value for the interaction between CU×ODD measures.

All results adjusted for the covariates SES, ethnicity, sex, presence of comorbidities other than ODD and number of CD symptoms.

*p includes Simes correction for multiple statistical tests.

R^2^ corresponds to the change between the model including the covariates and the model including the covariates plus the independent variable CU-raw score.

Additionally, the results of the GLM, adjusted by the covariates of the study, measuring the association of the CU raw score on the ODD measures also at age 3 for the children diagnosed with ODD at baseline (*n* = 61) showed that CU levels did not achieved significant contribution on the ODD level (p ≥ .810; R^2^ ≤ .006).

### Stability of ODD and callous-unemotional traits from age 3 to 5

The stability measures of ODD and CU between ages 3 and 5 yielded significant (*p* < .001) but moderate-low coefficients: intra-class correlation was .31 for callousness, .40 for uncaring, .03 for unemotional, .40 for total score and .42 for number of ODD symptoms. The presence of ODD (present/absent) obtained a Cohen’s kappa equal to κ = .35 between ages 3 and 4, and κ = .45 between ages 4 and 5.

### Association between CU and ODD at age 3 with psychological measures at age 5

Table 3 includes the GLM and logistic regressions for the total sample (*n* = 565 remaining participants in the second year of follow-up), entering the independent variables (CU and ODD measures) registered at age 3 and the dependent variables (clinical measures) at age 5 (except for social cognition, which was registered at age 4). Models were adjusted by the covariates of the study. This table provides the specific predictive capacity of CU and ODD at age 3 on the subjects’ clinical state two years later. Since one interaction parameter CU×ODD achieved significant results (social cognition, *p* = .05), single effects were estimated and interpreted (for children with ODD = absent, ODD = present, low CU score-percentile 25 of the distribution- and high CU score-percentile 75): high CU raw scores were associated with high scores in social cognition difficulties at age 4, but only for children without ODD at age 3. The other interaction parameters were excluded from the final models due to the lack of statistical significance. High CU levels at age 3 were predictive of higher levels of CU traits (callousness, uncaring, unemotional, total), a higher number of ODD symptoms, CAS total aggression, relational aggression, CBCL emotionally withdrawn, aggressive behavior, internalizing, externalizing and total scores, lower scores in functional impairment and high risk of use of services. The presence of ODD at age 3 was only predictive of a higher number of ODD symptoms (total and in both dimensions), lower scores in functional impairment and a higher risk of other comorbid disorders two years later.

**Table 3:** Association of the CU and ODD measured at age 3 on the psychological outcomes registered at age 5 (total sample, n = 565).

Dependent variables	^1^CU×	Independent variables (entered simultaneously)												*R* ^2^
	ODD	CU raw score						Oppositional defiant disorder (ODD)						
	*p*	B	SE		95%CI (B)		* *p*	B	SE		95%CI (B)		* *p*	
*ICU Callous-unemotional*														
Callousness	.500	0.104	0.018		.069	.139	**< .001**	0.20	0.535		-.847	1.254	.704	.080
Uncaring	.066	0.169	0.023		.124	.214	**< .001**	0.74	0.871		-.972	2.450	.397	.095
Unemotional	.879	0.082	0.017		.048	.115	**< .001**	0.55	0.548		-.529	1.623	.318	.053
Total	.258	0.355	0.045		.267	.443	**< .001**	1.51	1.622		-1.672	4.701	.351	.122
*DSM-IV quantitative*														
Number ODD symptoms	.311	0.018	0.007		.005	.032	**.014**	1.22	0.316		.602	1.843	**< .000**	.053
ODD-irritability dimension	.274	0.006	0.003		-.001	.013	.090	0.53	0.154		.224	.831	**.001**	.031
ODD-headstrong dimen.	.418	0.012	0.004		.004	.021	**.013**	0.60	0.187		.227	.963	**.002**	.045
*Aggression*														
CAS Aggression: total	.213	0.245	0.083		0.08;	0.41	**.003**	2.23	2.664		-3.00;	7.46	.561	.022
Relational aggression	.268	0.406	0.082		0.24;	0.57	**< .001**	1.79	3.082		-4.26;	7.85	.643	.047
** *Social cognition*														
SCDC: total (teacher)	.050													.080
ODD = No | CU = low		0.117^NO^	.021		0.08;	0.16	**< .001**	0.59^LOW^	.622		-0.64;	1.81	.346	
ODD = Yes | CU = high		0.009^YES^	.056		-0.10;	0.12	.870	0.82^HIGH^	.793		-0.74;	2.34	.302	
*CBCL/1½-5 scales*														
Emotionally reactive	.518	0.028	0.012		0.00;	0.05	**.042**	0.47	0.359		-0.24;	1.18	.447	.019
Anxious/depressed	.207	0.017	0.010		0.00;	0.04	.110	0.29	0.325		-0.35;	0.92	.665	.008
Somatic complaints	.413	0.011	0.007		0.00;	0.02	.164	0.42	0.243		-0.05;	0.90	.287	.010
Withdrawn	.375	0.015	0.007		0.00;	0.03	**.073**	-0.04	0.240		-0.51;	0.43	.858	.011
Sleep problems	.728	0.011	0.010		-0.01;	0.03	.244	0.25	0.359		-0.45;	0.96	.673	.003
Attention problems	.474	0.015	0.008		0.00;	0.03	.110	0.16	0.291		-0.42;	0.73	.691	.007
Aggressive behaviour	.575	0.058	0.023		0.01;	0.10	**.042**	1.96	1.038		-0.08;	4.00	.287	.019
Internalizing	.803	0.073	0.030		0.01;	0.13	**.016**	1.14	0.948		-0.72;	3.01	.229	.018
Externalizing	.516	0.073	0.028		0.02;	0.13	**.010**	2.12	1.209		-0.26;	4.49	.080	.018
Total	.802	0.181	0.077		0.03;	0.33	**.020**	4.02	2.830		-1.54;	9.58	.156	.016
*Functional impairment*														
CGAS: total score	.274	-0.151	0.037		-0.22;	-0.08	**< .001**	-5.28	1.250		-7.74;	-2.83	**< .001**	.050
*Categorical measures*	*p*	Callousness: raw score (CU)						Oppositional defiant disorder (ODD)						*R* ^2^
		B	SE	OR	95%CI (OR)		* *p*	B	SE	OR	95%CI (OR)		* *p*	
Comorbidity	.070	0.021	0.015	1.02	0.99;	1.05	.175	0.94	0.444	2.55	1.07;	6.10	**.050**	.019
Use of services	.910	0.052	0.012	1.05	1.03;	1.08	**< .001**	0.71	0.367	2.04	0.99;	4.19	.053	.080

^1^p-value for the interaction between CU×ODD measures

^NO^Single effects: contribution of CU raw score to the psychological measures for children without ODD (ODD = no).

^YES^Single effects: contribution of CU raw score to the psychological measures for children with ODD (ODD = yes).

^LOW^Single effects: contribution of ODD diagnosis to the psychological measures for children with low CU score (percentile 25).

^HIGH^Single effects: contribution of ODD diagnosis to the psychological measures for children with high CU score (percentile 75).

All results adjusted for the covariates SES, ethnicity, sex, presence of comorbidities other than ODD and number of CD symptoms.

*p includes Simes correction for multiple statistical tests.

**Registered at age 4 (teachers’ report).

R^2^ corresponds to the change between the model including the covariates and the model including the covariates plus the independent variable CU raw score.

For the ODD subsample at baseline, high CU levels at age 3 were only predictive of high risk of comorbid disorders at age 5 (B = 0.226, SE = 0.089, OR = 1.25, 95%CI: 1.05 to 1.50, *p* = .014, ΔR^2^ = .141).

## Discussion

The purpose of the present study was to evaluate the specific contribution of both CU traits and ODD, cross-sectionally and longitudinally, to several psychological characteristics in a large community sample of preschoolers. Controlling strictly for SES, ethnicity, sex, presence of CD symptom severity and other comorbidity, CU traits and ODD in preschoolers from the general population make an independent contribution to the outcomes studied. As a whole, these results indicate that children with ODD and high CU level traits present more severe clinical characteristics. Longitudinally, both conditions at age 3 are predictive of the continuity of oppositional symptomatology and a worse outcome two years later, while high levels in CU traits contribute to a wider variety of negative odds. CU traits were moderately stable from age 3 to 5. These results underscore the need for early identification of CU traits and the preventive potential of early recognition of these traits. Though children scoring high on CU traits show poor response to treatment, some recent intensive programs have proved effective [48], and interventions involving, for example, emotion recognition training, have resulted in significant improvements in affective empathy and conduct problems in children with high CU traits [49]. Early detection and intervention may be clinically useful.

We wanted to know whether the presence of ODD and the CU levels were associated with different clinical characteristics, or with different intensities, as early as preschool age. For most of the variables, CU levels and ODD had an independent effect cross-sectionally and longitudinally (only one interaction was significant), which means that both conditions merit independent clinical attention. Cross-sectionally, both were associated with higher ODD symptoms, externalizing and total symptoms, higher impairment, comorbidity and use of services. Also, high CU traits controlling for the presence of ODD were associated with higher aggression and withdrawn behavior, whereas ODD controlling for the presence of high CU traits was related to higher emotional reactivity and anxiety. In particular, the association of CU traits and withdrawn behaviors, which may be manifested by avoidance of eye contact, not being involved with others, lack of interest, etc., may be in line with recent results indicating that children with ODD and high levels of CU showed low levels of eye contact toward their mothers [20], and that impaired eye contact is a unique characteristic of children with CU traits [50]. Our results are also in agreement with the strong association between CU and behavior problems found in several samples [7] and between ODD and emotional reactivity [51].

Along similar lines, with regard to the predictive potential of ODD and CU traits we found that both conditions are associated at follow-up (age 5) with poorer prognosis. Specifically, if both levels were at high at age 3 they predicted a higher number of ODD symptoms and worse functional impairment at age 5. Considering CU and ODD predictions independently, controlling for each other, higher CU when the child was 3 was a risk factor for numerous and clinically relevant outcomes (persistence of CU traits, higher scores in aggression measures, higher symptomatology, greater impairment and more service use), whereas ODD at age 3, controlling for CU traits, was predictive at age 5 of comorbidity and worse functioning. Greater knowledge about the characteristics of children with CU traits not associated with conduct problems is seen as a priority by researchers in this field [14]. Our results regarding CU are in line with those described in older children and adolescents with CU traits [7, 52].

CU traits at age 3 predicted ODD (particularly the headstrong component) at age 5, suggesting that children with cold, non-empathic, and uncaring traits are likely to show behavioral symptoms of oppositionality. However, the association was not bidirectional and ODD at age 3 did not predict CU behaviors. The lack of bidirectionality may be indicating the differential conceptualization of both constructs: CU traits as temperamental/personality vulnerability traits and ODD as a possible resultant behavior associated with these traits. An aspect especially worthy of note was related to predicting CU-only traits over time. Few cases receive clinical attention because of lack of empathy or lack of guilt, and even fewer in the preschool years. Therefore, the detection of these characteristics early in life is of clinical value in prevention and re-education in relation to prosocial behavior, perspective-taking, unacceptability of aggressive behavior, affective reasoning, ability to make associations between negative and positive consequences, and other difficulties typically described in children with CU traits.

Regarding the stability of the CU traits, our results indicate low to moderate stability. Previous analyses with preschoolers over a period of 6 months to 2 years (see the review on this topic by [4]) yielded an estimated mean of *r* = .59. Our study differs from the studies reviewed there in relation to the timeframe, the coefficients provided and the informants. In those studies it was the parents who reported, while in ours it was the teachers; also, CU traits were assessed for most of the children by different teachers, and this introduces more variability in the assessment. Importantly, the stability of CU traits that we found was comparable to the stability of ODD symptoms. Given the marked developmental changes that can occur at these early ages, when empathy is developing, the stability of CU difficulties is noteworthy. With regard to the lack of correlation between ages 3 and 5 for the unemotional component of the ICU, Roose et al. [53] noted that some of the items on the unemotional scale reflect emotional expression that appears to be independent of antisocial behavior, and its usefulness in this questionnaire must consequently be reconsidered. Recently, Ray et al. [54] studying a sample of adolescents, have pointed out that the unemotional scale contributes to an overall CU factor but is weakly associated with other subscales; they recommend further research to conceptualize it within the broader construct of CU behaviors.

The study has several positive aspects. First, ODD was studied separately from CD, which enabled us to identify the specific clinical characteristics of ODD when in combination with CU. Second, the analyses controlled for the presence of conduct disorder severity. Third, the population addressed was represented by a large sample of preschool children with good potential for preventive intervention. Fourth, dimensional and interview-based categorical data were available for defining ODD and controlling for comorbid psychopathology. And fifth, we employed a widely-used instrument to assess callous-unemotional traits, which permits comparison of these results with those from other studies of older children. Furthermore, we used different informants (parents and teachers), who could observe the child in different situations. However, some limitations should also be taken into account when interpreting the present results. We studied a very young sample of the general population, and psychopathology is not very frequent in community samples; this could have affected the emergence of more associations. CU traits are moderately stable from childhood to adolescence [4], but more and longer studies are needed in relation to stability from preschool age, a developmental period when aggressive behavior is prominent and guilt and empathy begin to emerge [55].

Taken together, the results also suggest that the assessment and identification of CU traits from preschool age onwards might help us to identify a subset of children who may have sustained and severe social and behavioral problems. The early identification of these traits can permit modification and re-education in relation to difficulties with affective experience. These results may also be useful for future nosological classifications.

## Supporting Information

S1 Table ODD items used in the study. (DOCX)
