# Supplementary material for: Clinical Characteristics of Preschool Children with Oppositional Defiant Disorder and Callous-Unemotional Traits
Source: PLoS One. 2015 Sep 29;10(9):e0139346. doi: 10.1371/journal.pone.0139346 (PMC4587853; doi:10.1371/journal.pone.0139346)
Supplement: S1 Table — (DOCX) [file pone.0139346.s001.docx]

S1 Table. *ODD items used in the study.*

| Item | Content |
| --- | --- |
| SDQ^3-4 Conduct^ 5 | Temper tantrums or hot temper |
| SDQ^3-4 Conduct^ 7 | Obedient, does what adults request |
| SDQ^3-4 Conduct^ 18 | Argumentative with adults |
| SDQ^3-4 Conduct^ 22 | Spiteful to others |
| *I-A* | Deliberately annoys others |
| *I-A* | Blames others for his/her mistakes |
| *I-A* | Easily offended by things others say |
| *I-A* | Angry/resentful |

SDQ^3-4 Conduct^ <http://www.sdqinfo.com/>

I-A: item added for the study.
